# Hybridization alters the gut microbial and metabolic profile concurrent with modifying intestinal functions in Tunchang pigs

**DOI:** 10.3389/fmicb.2023.1159653

**Published:** 2023-04-20

**Authors:** Jiayi He, Yunchao Zhang, Hui Li, Yanshe Xie, Guiqing Huang, Chen Peng, Pengju Zhao, Zhengguang Wang

**Affiliations:** ^1^Hainan Institute of Zhejiang University, Sanya, China; ^2^College of Animal Science, Zhejiang University, Hangzhou, China; ^3^Long Jian Animal Husbandry Company, Haikou, China

**Keywords:** gut microbiome, gut metabolome, intestinal function, mammalian hybridization, wild boar

## Abstract

**Introduction:**

Hybridization has been widely used among Chinese wild boars to improve their growth performance and maintain meat quality. Most studies have focused on the genetic basis for such variation. However, the differences in the gut environment between hybrid and purebred boars, which can have significant impacts on their health and productivity, have been poorly understood.

**Methods:**

In the current study, metagenomics was used to detect the gut microbial diversity and composition in hybrid Batun (BT, Berkshire × Tunchang) pigs and purebred Tunchang (TC) pigs. Additionally, untargeted metabolomic analysis was used to detect differences in gut metabolic pathways. Furthermore, multiple molecular experiments were conducted to demonstrate differences in intestinal functions.

**Results:**

As a result of hybridization in TC pigs, a microbial change was observed, especially in *Prevotella* and *Lactobacillus*. Significant differences were found in gut metabolites, including fatty acyls, steroids, and steroid derivatives. Furthermore, the function of the intestinal barrier was decreased by hybridization, while the function of nutrient metabolism was increased.

**Discussion:**

Evidences were shown that hybridization changed the gut microbiome, gut metabolome, and intestinal functions of TC pigs. These findings supported our hypothesis that hybridization altered the gut microbial composition, thereby modifying the intestinal functions, even the host phenotypes. Overall, our study highlights the importance of considering the gut microbiome as a key factor in the evaluation of animal health and productivity, particularly in the context of genetic selection and breeding programs.

## 1. Introduction

Mammals have complicated intestinal microorganisms, which play a critical role in a variety of physiological processes, including nutrient metabolism and absorption (Meng et al., 2020), immune response (Ivanov et al., 2009), and growth performance (Zhou et al., 2021). Furthermore, many studies had shown that intestinal microorganisms were altered by host genome, breed age, sex, maternal effect, and diets (Adhikari et al., 2019; Bergamaschi et al., 2020). From a genetic point of view, the approximate Bayesian computation analysis of 103 genomes of Asian and European wild boar and domestic pigs demonstrated the existence of gene flow during and after domestication (Frantz et al., 2015). Thus, it was feasible to suspect the hybridization would alter the intestinal environment of wild boar. The current study identified that BT pigs had greater growth performance than TC pigs, while the meat quality was maintained (Wang et al., 2011). However, the differential intestinal microbiome between TC pigs and BT pigs was still unknown, let alone the alteration of host–microbiota interaction by the hybridization.

Recent studies indicate that the intestinal microbiota plays a vital role in the adaptive evolution of mammal species (Moeller and Sanders, 2020). Furthermore, the mammalian intestinal metabolomes mirror microbiome composition and host phylogeny (Gregor et al., 2022). Therefore, a comprehensive analysis was needed in order to reflect the differences between purebred pigs and crossbred pigs. For instance, tracking the microbial and metabolic trends over time could clarify the colonization of bacteria and their effects (Wang et al., 2019). In addition, permutational multivariate analysis of variance was used to calculate whether interfering factors affect the microbiome and metabolome. Recent studies of correlation analysis also provided a perspective to understand the connection between microbiome and metabolome. Such analysis may explain how hybridization alters the intestinal microbiome. Finally, a variety of strategies were used to compare the intestinal functions between TC pigs and BT pigs. The expression of intestinal functional-related mRNAs was related to the intestinal state and reflected the main functions (Moran et al., 2010; Hartmann et al., 2016; Brooks II et al., 2021). On the other hand, intestinal histology indicated the capability of nutrient absorption (Wang et al., 2019), and serum LPS levels were used to reveal the intestinal barrier function (Ghosh et al., 2020) because serum LPS was now an accepted surrogate marker for assessing *in vivo* intestinal permeability.

To complement these blank studies, we designed an experimental approach that compares differential intestinal environments in certain aspects. First, a comparison with mRNA expression data, intestinal histology analysis, and serum lipopolysaccharide level revealed a significant difference in intestinal functions between TC pigs and BT pigs. In addition, collecting the intestinal content by rectum stimulus at the pre-weaning stage (PW, 30 days of age), weaned stage (WD, 60 days of age), and growth stage (90 days of age) to explore the colonization process of bacteria, and the changes of metabolic pathway. Moreover, the interfering factors, such as diet and pigpen, were strictly controlled, using a permutational multivariate analysis to calculate the influences of sex and maternal effect. Correlation analysis was also used to reflect the potential mechanism of host–microbiota interactions. In summary, the hybridization improved the nutrient metabolism and absorption functions but decreased the intestinal barrier function.

## 2. Materials and methods

### 2.1. Pigs and experimental design

A total of 18 healthy piglets (TC pigs, *n* = 9; BT pigs, *n* = 9) born from nine sows were selected and raised on a local commercial farm (Tunchang, China). Piglets lived with their mothers for 1 month of adaptation to solid feed and would imitate their mothers’ behavior to eat solid feed; nevertheless, it was still hard to collect their intestinal content before 30 days old because they ate very little. Subsequently, piglets (30 days old) started weaning and housed by species. All pigs were provided with a commercial diet with *ad libitum* access to clean water. The growth performance parameters (body weight and average daily gain) were measured monthly from birth.

### 2.2. Sample collection

At the pre-weaning stage (PW, 30 days of age, *n* = 18), weaned stage (WD, 60 days of age, n = 18), and growing stage (GT, 90 days of age, *n* = 18), fresh intestinal content samples were collected by rectal stimulation of TC pigs and BT pigs. Each sample was stored in a 50 ml sterile centrifuge tube and kept on ice during transportation. The body weight was measured to the nearest 0.1 kg monthly since birth, and average daily gain was monitored. At 90 days of age, three pigs were randomly selected from TC pigs and BT pigs, respectively, and were slaughtered. Blood was drawn from the anterior vena cava and centrifuged at 3,000 rpm for 15 min at 4°C. Plasma was collected and immediately stored at −80°C for further analysis. Tissue samples were collected from the midsection (4 cm) of the duodenum, jejunum, ileum, and colon. After that, all samples were stored in a − 80°C freezer for cryopreservation.

### 2.3. DNA extraction and whole-genome shotgun sequencing

DNA extraction and shotgun metagenomic sequencing were conducted at Personal Biotechnology Co., Ltd. (Shanghai, China). Total microbial genomic DNA in the intestinal content of 54 samples was extracted by a DNeasy PowerSoil Kit (QIAGEN, Hilden, Germany), following the manufacturer’s instructions. The quality and quantity of the extracted DNA were assessed by agarose gel electrophoresis and a NanoDrop ND-1000 spectrophotometer (Thermo Fisher Scientific, Waltham, MA, United States). The qualified DNA was processed to construct the shotgun metagenomic sequencing library by a TruSeq DNA Nano High-Throughput Library Preparation Kit (Illumina, San Diego, CA, United States). The sequencing strategy was paired-end 150 bp reads with an insert size of 400 bp. A dual-indexed barcode structure was applied for multiplexing, and 1% PhiX Control v3 was added to the library for quality monitoring. The prepared libraries were stored at – 20°C before sequencing. The sequencing platform was Illumina NovaSeq (Illumina, San Diego, CA, United States). The cluster density was in the range of 1,255–1,412 K clusters/mm2, and the error rate was <0.05% for the sequencing run.

### 2.4. Metagenomics data assembly and analyses

Raw sequenced reads were first processed to obtain high-quality clean reads. Adapter sequences were removed by Cutadapt (v1.2.1) (Martin, 2011), and raw reads were processed by a 5-bp sliding window to trim low-quality sequences (< Q20, read accuracy <99%). Trimmed reads with a length of >50 bp and no ambiguous bases were kept for further analyses. Human reads were removed by KneadData (v0.9.0) and BMTagger (v3.101). The clean reads were assembled by MEGAHIT (v1.0.5) with a succinct de Bruijn graph approach (Li et al., 2015). The coding sequences (CDS, > 300 bp) were predicted by MetaGeneMark (v3.25; Zhu et al., 2010). CDSs were clustered by CD-HIT (v4.8.1; Fu et al., 2012) at 90% amino acid sequence identity to obtain a non-redundant gene catalog. The abundance of genes was calculated as the number of aligned reads by SOAPdenovo2 (v1.0) (Luo et al., 2012). The taxonomy was annotated by searching against the NCBI-NT database by BLASTN (e-value <0.001) and annotated by MEGAN with the lowest common ancestor approach (Huson et al., 2007). The functional gene was annotated by searching the sequence of the non-redundant genes against the KEGG databases (release 90.0) by DIAMOND protein aligner (v2.0.4), with an e-value of <0.001 and coverage ratio of >40% (Buchfink et al., 2015). Microbial compositional variation (beta diversity) was calculated by Bray–Curtis distance metrics and visualized by principal coordinate analysis (PCoA) and non-metric multidimensional scaling (NMDS) hierarchical clustering (Bray and Curtis, 1957; Ramette, 2007). Permutation analysis (999 permutations) was conducted for microbial taxonomic composition between TC pig and BT pig samples by the Adonis function in R (v4.1.0). The bacterial co-correlation matrix was calculated by the R “igraph” package, and figures of the co-correlation network were plotted by Gephi. R (v4.1.0) was used throughout the study for data processing, analysis, and visualization (Caporaso et al., 2010).

### 2.5. Sample preparation for liquid chromatography–tandem mass spectrometry (LC–MS) analysis

The extraction of intestinal microbiota metabolites was performed with minor modifications, as described earlier (Turroni et al., 2016). In brief, approximately 1.0 g of intestinal content samples were mixed with 600 μL of MeOH [stored at −20°C, containing 2-Amino-3-(2-chloro-phenyl)-propionic acid (4 ppm)]. After vortex mixing for 30 s, samples were placed in a tissue grinder for 90 s at 60 Hz, with the addition of 100 mg of glass bead followed by ultrasound at room temperature for 10 min. Finally, the samples were centrifuged at 12,000 rpm for 10 min at 4°C, and the supernatant was collected for an LC–MS analysis after filtering through a 0.22 μm filter.

### 2.6. LC–MS analysis

Untargeted intestinal metabolomics was performed using an LC–MS platform at Personal Bio Inc. (Shanghai, China). The LC analysis was performed on a Vanquish UHPLC System (Thermo Fisher Scientific, United States). Chromatography was carried out with an ACQUITY UPLC ® HSS T3 (150 × 2.1 mm, 1.8 μm) (Waters, Milford, MA, United States). Mass spectrometric detection of metabolites was performed on Q Exactive HF-X (Thermo Fisher Scientific, United States) with an ESI ion source. Simultaneous MS1 and MS/MS (full MS-ddMS2 mode, data-dependent MS/MS) acquisition were used.

### 2.7. LC–MS data processing and multivariate analysis

The raw data were first converted to mzXML format by MSConvert in the ProteoWizard software package (v3.0.8789) and processed using XCMS (Sud et al., 2007) for feature detection, retention time correction, and alignment. The metabolites were identified by accuracy mass (< 30 ppm) and MS/MS data which were matched with HMDB (Abdelrazig et al., 2020),^1^ MassBank (Gagnebin et al., 2017),^2^ LIPID MAPS (Thévenot et al., 2015),^3^ mzCloud (Xia and Wishart, 2011),^4^ and KEGG (Dunn et al., 2011).^5^ . QC robust LOESS signal correction (QC-RLSC; Want et al., 2013) was applied for data normalization to correct any systematic bias. After normalization, only ion peaks with relative standard deviations (RSDs) of less than 30% in QC were kept to ensure proper metabolite identification.

The ropls (Boulesteix and Strimmer, 2007) software was used for all multivariate data analyses and modelings. Data were mean-centered using scaling. Models were built on orthogonal partial least-square discriminant analysis (OPLS-DA) and partial least-square discriminant analysis (PLS-DA). The metabolic profiles could be visualized as a score plot, where each point represents a sample. The corresponding loading plot and S-plot were generated to provide information on the metabolites that influence the clustering of the samples. All the models evaluated were tested for overfitting with methods of permutation tests. The descriptive performance of the models was determined by R2X (cumulative) (perfect model: R2X (*cum*) = 1) and R2Y (cumulative) (perfect model: R2Y (*cum*) = 1) values, while their prediction performance was measured by Q2 (cumulative) (perfect model: Q2 (*cum*) = 1) and a permutation test. The permuted model should not be able to predict classes. The R2 and Q2 values at the Y-axis intercept should be lower than those obtained from the non-permuted model for Q2 and R2. OPLS-DA allowed the determination of discriminating metabolites using the variable importance in projection (VIP). The *p*-value, VIP produced by OPLS-DA, and fold change (FC) were applied to discover the contributable variable for classification. Finally, the *p*-value of <0.05 and the VIP values of >1 were considered to be statistically significant metabolites.

Differential metabolites were subjected to pathway analysis by MetaboAnalyst (Trygg and Wold, 2002), which combines results from powerful pathway enrichment analysis with the pathway topology analysis. The identified metabolites in metabolomics were then mapped to the KEGG pathway for biological interpretation of higher level systemic functions. The metabolites and corresponding pathways were visualized using the ggplot software.

### 2.8. Detection of mRNA expression

Total RNA Isolation Kit (SparkJade, China) was used to extract RNA from the jejunum, the ileum, and the colon tissues. A cDNA library was prepared by transcribing 2 μg of RNA with the SPARK script II 1st Strand cDNA Synthesis Kit (SparkJade, China) and qPCR performed with specific primer pairs (Supplementary Table S6) and the 2 × SYBR Green qPCR Mix (SparkJade, China) on the CFX96 system (Bio-Rad, United States). Relative expression levels were calculated using the 2^-△△Ct^ method, and *β-actin* was utilized to normalize the relative mRNA expression levels of the target genes. Student’s *t*-test was used to evaluate the statistical difference of each target gene.

### 2.9. Intestinal histology analysis

Approximately 4 cm of each sample was taken from the middle sections of the duodenum, jejunum, and ileum. These tissue samples were washed with cold sterile saline and immediately fixed in 4% paraformaldehyde solution (Biosharp, China) for 24 h followed by dehydrating and embedding in paraffin wax before transverse sections were cut. The preserved samples were stained with hematoxylin and eosin according to the manufacturer’s guidelines of the Hematoxylin–Eosin (HE) Stain Kit (Solarbio, United States). A total of 12 well-orientated sections of villi and their adjoint crypts in each sample were performed using a Nikon A1 inverted laser scanning confocal microscope (Nikon, Japan). Images were analyzed using Image-Pro Plus software (version 6.0, Media Cybernetics, United States).

### 2.10. Serum lipopolysaccharide levels

Serum LPS levels were measured using ELISA Kit (JingMei Biotechnology, China), and the product was visualized at 450 nm in a microplate reader (BioTek Synergy HT microplate reader, United States). Student’s *t*-test was used to evaluate the statistical difference.

### 2.11. Correlation analysis between microbiome and metabolome

Correlation analysis between the microbiome and metabolome was calculated by Spearman’s correlation coefficient and *p*-value and plotted by the R “pheatmap” package. Bacteria were deduced from the top 20 genera which accounted for more than 80% of the microbial sequence reads, and metabolites were deduced from the differential KEGG metabolic pathways.

## 3. Results

### 3.1. Hybridization improves the nutrient metabolism of TC pigs

**Figure 1:**
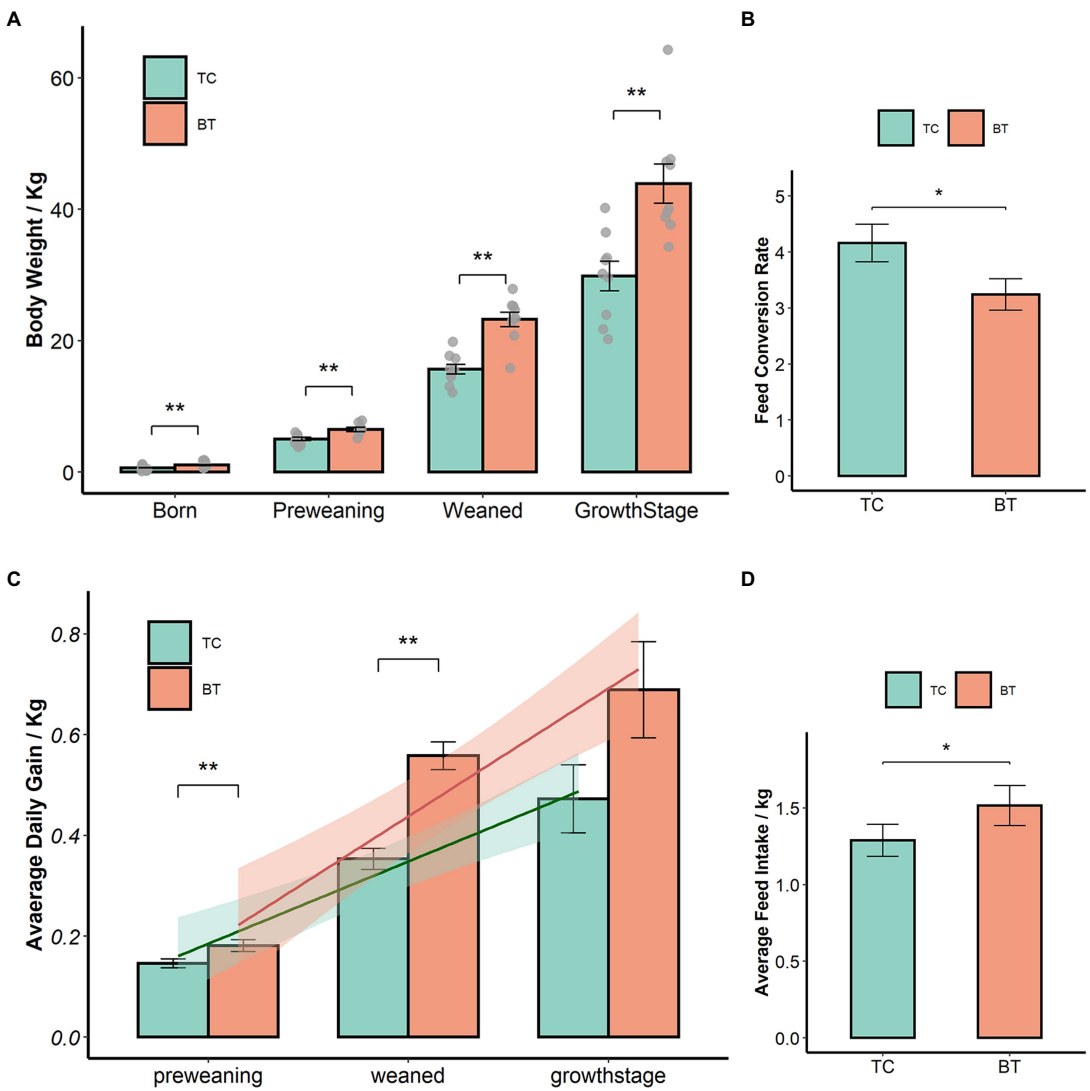
Hybridization improved the growth performance of TC pigs, the values showed in the bar presented as means ± SEM, and differences in the figures were marked by “*” and “**” while *p* < 0.05 and *p* < 0.01, respectively. **(A)** Body weight. **(B)** Average daily gain. **(C)** Feed conversion rate. **(D)** Feed intake.

**Figure 2:**
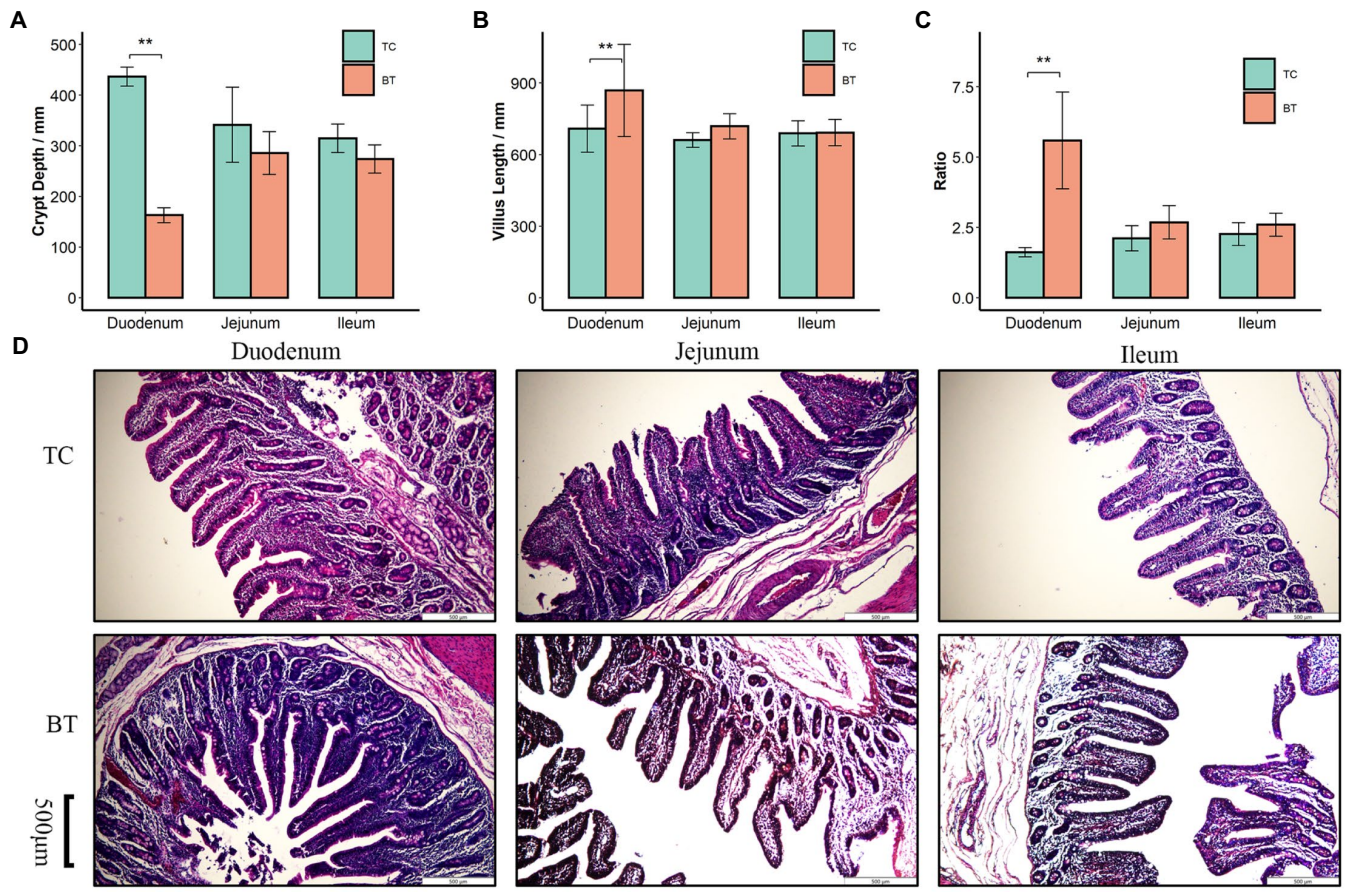
Histological analysis of the duodenum, jejunum, and ileum in TC pigs and BT pigs, the values showed in the bar presented as means ± SEM, and differences in the figures were marked by “*” and “**” while *p* < 0.05 and *p* < 0.01, respectively. **(A)** Crypt depth. **(B)** Villus length. **(C)** The ratio of villus length to crypt depth. **(D)** HE staining for histological examination.

We confirmed that the hybridization significantly improved (*p* < 0.05) the body weight of TC pigs (Figure 1A), which were healthy throughout the feeding trial period. The average daily gain and feed intake were increased as well (Figures 1B,D). In addition, the feed conversion rate was decreased (Figure 1C). Intestinal histology is critical to maintaining ecosystem stability and performance. However, only the histology of the duodenum was changed (Figures 2A–D) by hybridization, indicating the histology was similar in TC pigs and BT pigs overall. As a result, the differential growth performance may be caused by the intestinal functions and intestinal microbiota.

**Figure 3:**
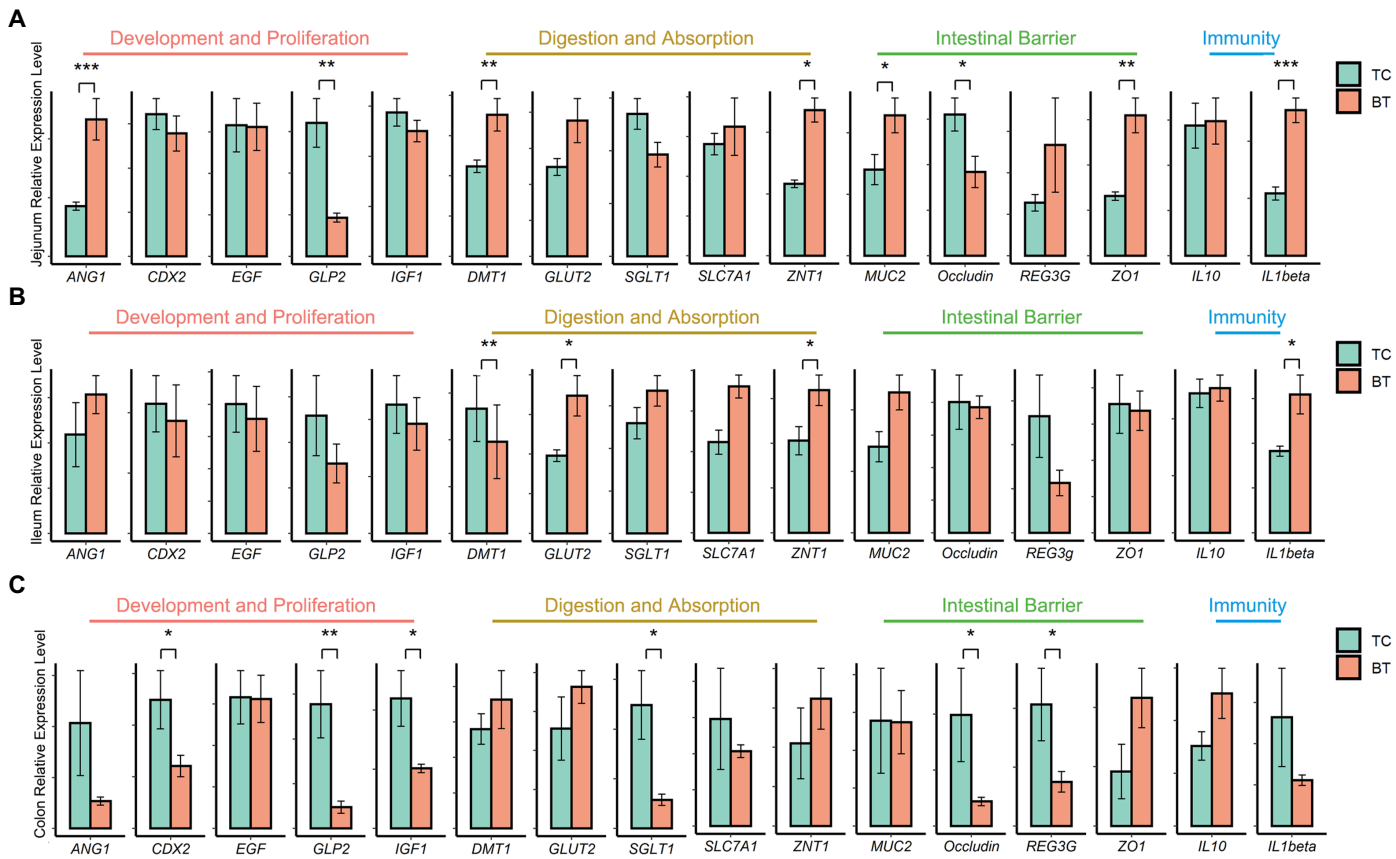
mRNA expression of genes related to intestinal functions was assessed by real-time PCR and normalized to beta-actin. The values showed in the bar are presented as means ± SEM, and differences in the figures were marked by “*” and “**” while *p* < 0.05 and *p* < 0.01, respectively. **(A)** mRNA expression in the jejunum tissue. **(B)** mRNA expression in the ileum tissue. **(C)** mRNA expression in the colon tissue.

Then, we assayed mRNA expression in different intestine segments of TC pigs and BT pigs, to compare the differences in intestinal functions. We examined mRNA expression of intestinal development and proliferation was downregulated (*p* < 0.05) in the colon after hybridization (Figure 3C), and it was related to the colonization of intestinal microbiota. In addition, the mRNA expression of nutrient metabolism and absorption was upregulated (*p* < 0.05) in the small intestine by hybridization (Figures 3A,B), including zinc transporter1 (ZNT1) and glucose transporter 2 (GLUT2). These transporters may contribute to the greater growth performance of BT pigs.

### 3.2. Hybridization decreases the intestinal barrier function of TC pigs

**Figure 4:**
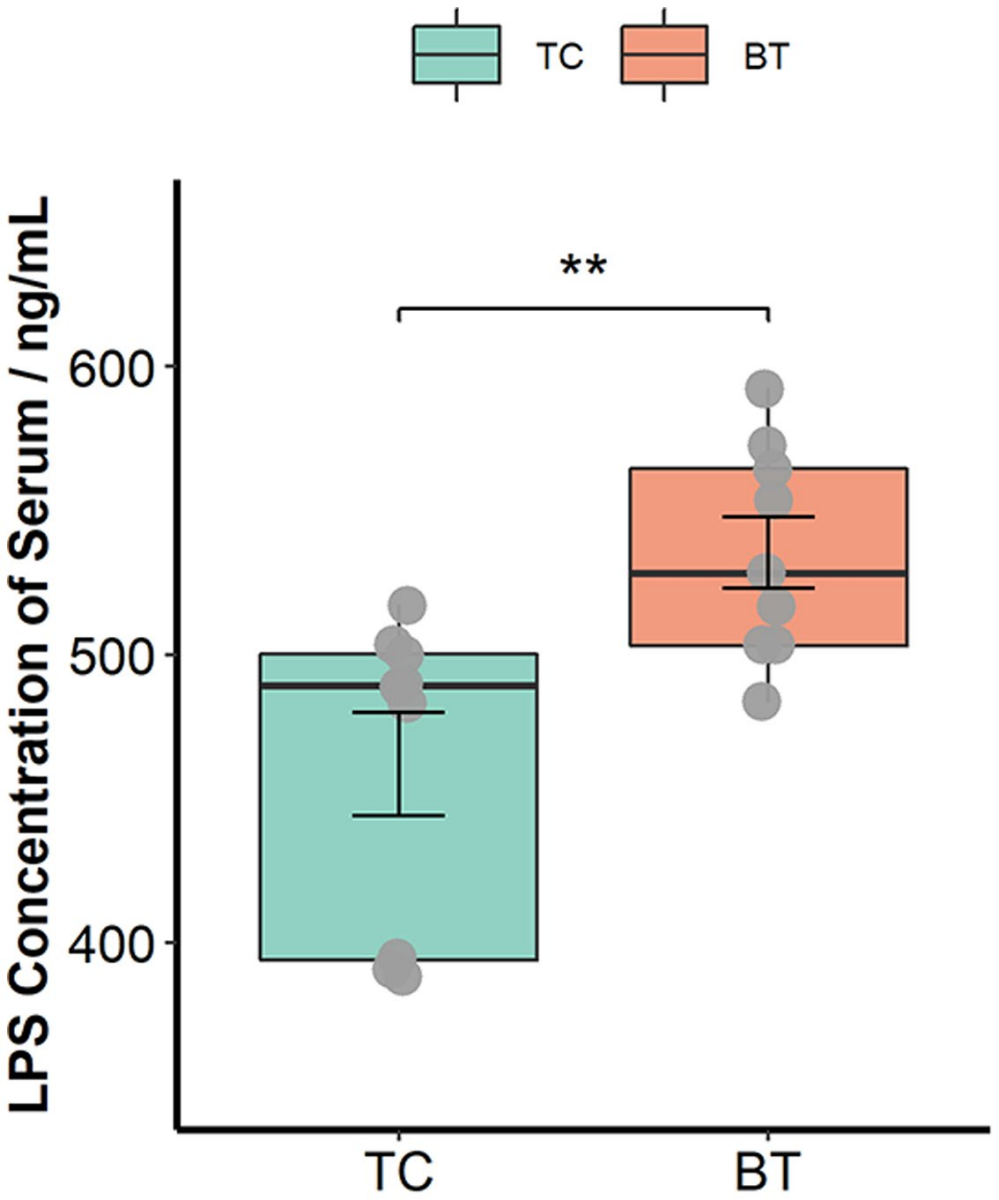
LPS concentration of serum was assessed by the ELISA Kit. The values showed in the boxplot presented as means ± SEM, and differences in the figures were marked by “*” and “**” while *p* < 0.05 and *p* < 0.01, respectively.

The intestinal barrier plays an important role in mammalian metabolism and immunity. Remarkably, mRNA expression of the intestinal barrier was downregulated in the colon after hybridization, including regenerating family member 3 gamma (REG3G) and occludin (Figure 3C). Specifically, REG3G had both bacteriostatic and bactericidal activities, and occludin was positively correlated with intestinal permeability. Furthermore, the serum LPS concentration was higher after hybridization (Figure 4). This evidence showed that the ability of anti-inflammation was impaired by the hybridization of TC pigs.

### 3.3. Microbial community richness was similar, but the diversity was different between TC pigs and BT pigs

**Figure 5:**
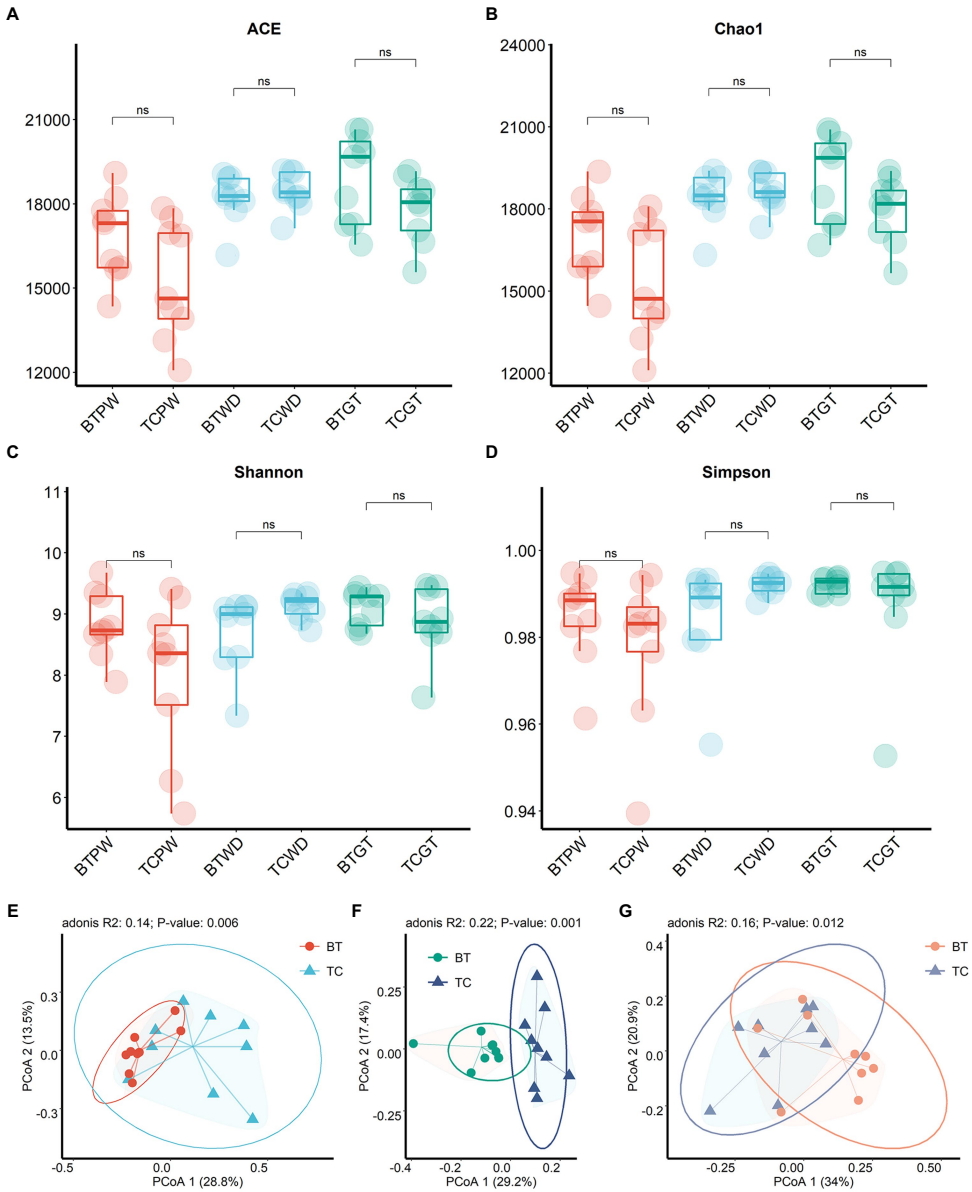
Microbial community richness and diversity between TC pigs and BT pigs at different stages. **(A)** ACE index. **(B)** Chao1 index. **(C)** Shannon index. **(D)** Simpson index. **(E–G)** Principal coordinate analysis (PCoA) was calculated from metagenome sequence data from individual samples, which showed the differences in microbiota composition over time.

To achieve comprehensive comparisons, we detected the intestinal microbiota through microbial richness and diversity. The current metagenomic analysis included a total of 54 samples collected longitudinally from nine TC piglets and nine BT piglets, obtaining an average of 46,007,294.19 reads per sample. Herein, taxonomic alpha diversity was estimated using Simpson, Chao1, ACE, and Shannon indices (Figures 5A–D). These indices showed no difference (*p* > 0.05) between TC pigs and BT pigs, reflecting that the numbers of observed taxa were similar. Principal coordinate analysis (PCoA, Figures 5E–G) based on the Bray–Curtis distance was performed to assess the beta diversity. Unlike alpha diversity, beta diversity demonstrated compositional differences in bacterial communities between TC pigs and BT pigs. Notably, the microbial compositional difference was most significant (*p* = 0.001) at the weaned stage. These data indicated that the microbial abundance of different taxa would be different after hybridization.

### 3.4. Microbial community dynamics and dominant microbiota were altered by age in TC pigs and BT pigs

**Figure 6:**
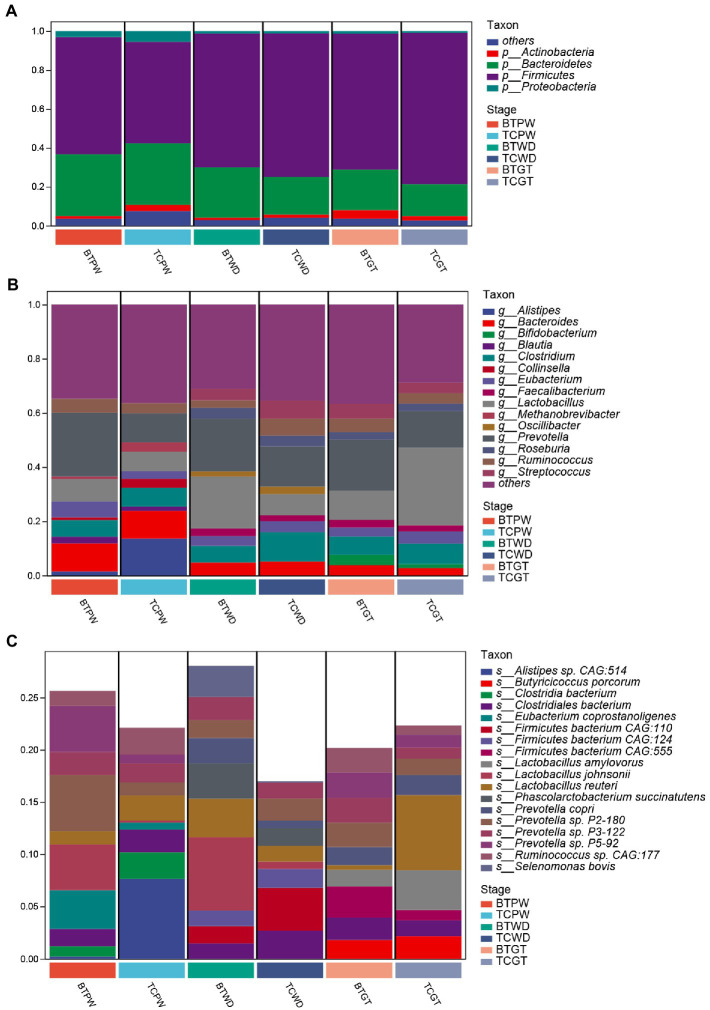
Composition and differences of the highly abundant microbial community at different stages in TC pigs and BT pigs. **(A)** Phylum-level, **(B)** genus-level, and **(C)** species-level composition of intestinal microbial communities; each bar shows the average relative abundance of the top 10 microbiota among the samples.

As shown in Figure 6A, the majority of phyla in all samples were *Firmicutes*, *Bacteroidetes*, and *Actinobacteria*. The detailed data are presented in Supplementary Table S1. Nevertheless, the abundance of *Bacteroides* was increased (*p* < 0.05) after hybridization at weaned stage (Supplementary Table S2). At the genus level, consistent stepwise increases or decreases were seen for all samples in most of the taxa from the pre-weaning stage to the growth stage (Figure 6B). However, *Lactobacillus*, by far the most differential genus, showed obvious differences in TC pigs and BT pigs, not only in the tendency but also in the relative abundance. Existing species-level data revealed such difference was led by *Lactobacillus reuteri*, *Lactobacillus johnsonii*, and *Lactobacillus amylovorus* (Figure 6C). Furthermore, the microbial composition was significantly different at the species level in TC pigs and BT pigs by stage (Supplementary Table S2), which was consistent with the beta diversity analysis.

**Figure 7:**
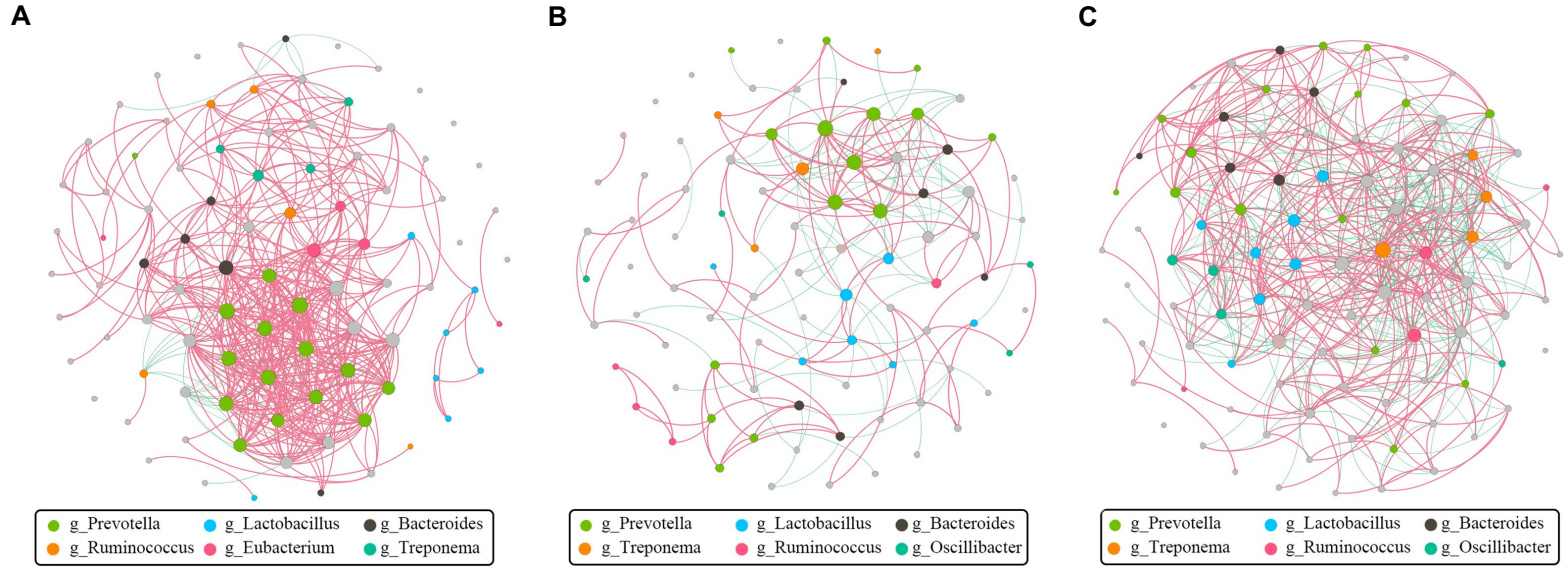
Co-correlation analysis of bacterial network by different stages. Each node represents a species, the size of each node is proportional to the relative abundance, and the color of the nodes indicates their taxonomic assignment. The red and green lines represent the positive and negative correlations, respectively. Only lines corresponding to correlations with a magnitude greater than 0.5 are shown. **(A)** Pre-weaning stage. **(B)** Weaned stage. **(C)** Growth stage.

The goal of network inference was to identify combinations of microorganisms that show significant co-correlation across species and to combine them into a network. Network analysis can also reveal why some microbial groups consistently occur together or whether certain microbial taxa are more important for maintaining network structure. Obviously, *Prevotella*, positively associated with many other bacteria, was the dominant and core genus at the pre-weaning stage (Figure 7A). Then, *Prevotella* and *Lactobacillus* showed numerous correlations at the weaned stage (Figure 7B), indicating their stability and importance under the weaning stress. Finally, the core genus of *Prevotella* was replaced by multiple genera at the growth stage as the network showed (Figure 7C), indicating the relative stability of intestinal microbiota. Above all, hybridization changed not only the microbial composition but also the microbial connections as well.

### 3.5. Predicted functions through the intestinal metagenome of TC pigs and BT pigs

**Figure 8:**
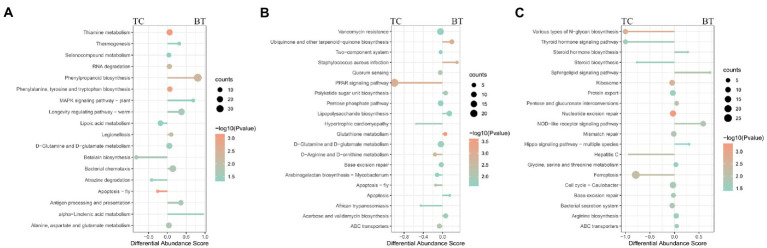
Enrichment analysis of KEGG pathway through the intestinal metagenome. Pathways were grouped by stages: **(A)** pre-weaning stage, **(B)** weaned stage, and **(C)** growth stage. Each point represents a pathway, the size of each point is proportional to the counts, and the color of the point indicates their *p*-values. The length of the line represents the differential abundance score (DAS) calculated by the relative gene ratio.

To compare the specific physiological properties between TC pigs and BT pigs, we next conducted KEGG analyses of the intestinal metagenome, which were identified from different stages. According to the KEGG analyses (Supplementary Table S3), hybridization improved the biosynthesis of many aromatic amino acids at the pre-weaning stage, such as phenylpropanoid, tyrosine, and tryptophan (Figure 8A). Intriguingly, these aromatic amino acids were involved in the biosynthesis of melanin. We suspected such aromatic amino acids affected the biosynthesis of melanin and coat color as the coat color of BT pigs with all-black while the coat color of TC pigs with piebald. Then, the enrichment of the ‘PPAR signaling pathway’ was detected to be decreased by hybridization (Figure 8B). The PPAR signaling was associated with lipid metabolism and adipocyte differentiation, but more evidence was needed to demonstrate its function. Finally, we detected that the ‘NOD-like receptor signaling pathway’ was enriched in BT pigs at the growth stage (Figure 8C), reflecting a bacterial infection caused by hybridization. At the same time, some pathways related to the construction of the intestinal barrier were decreased by hybridization. These data were consistent with the conclusion of intestinal functions.

### 3.6. Major metabolites of TC pigs and BT pigs at different stages

**Figure 9:**
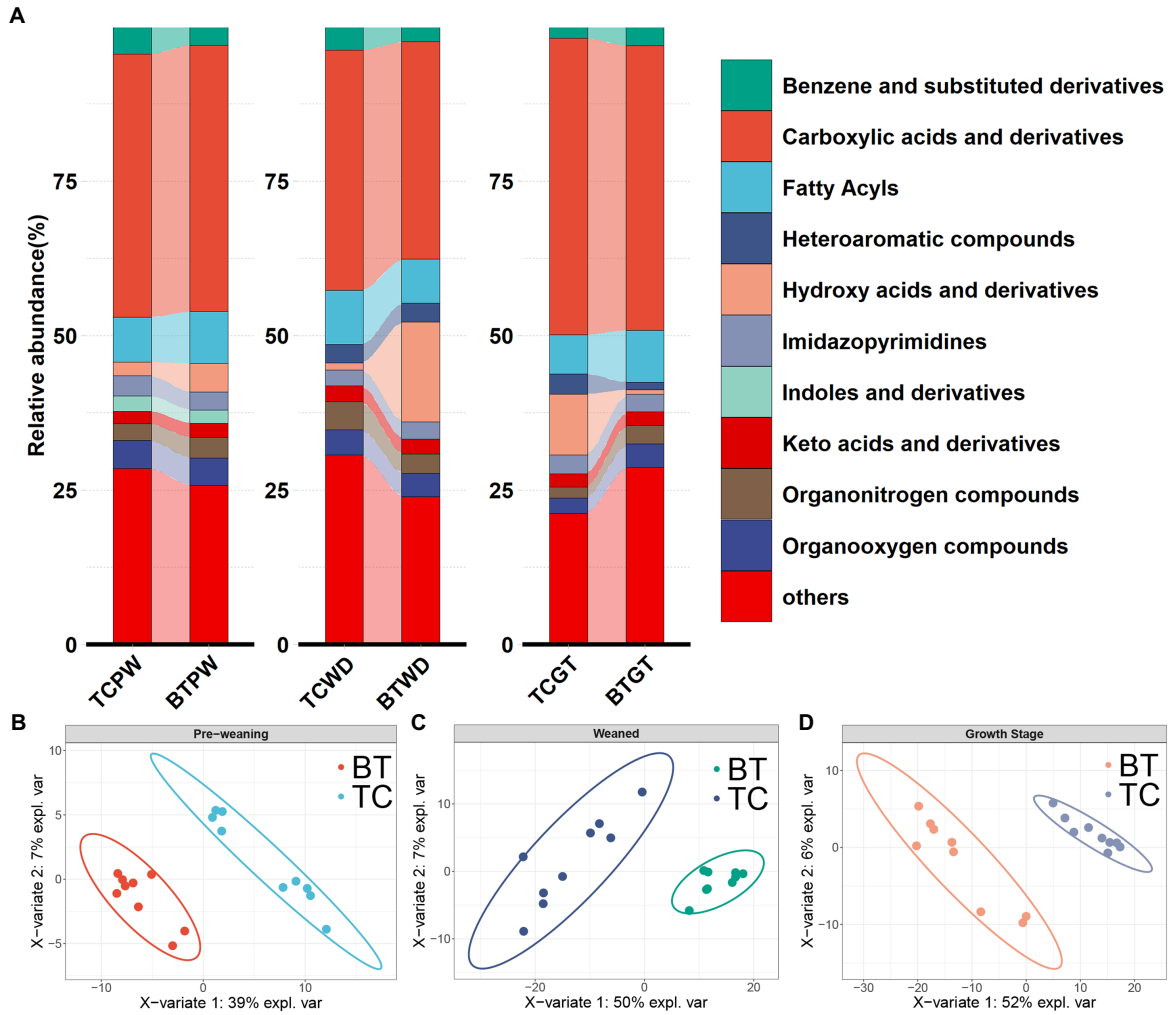
Metabolic composition and pattern were altered by hybridization. **(A)** Intestinal metabolite class composition according to relative metabolite masses (sum of standardized abundances), metabolites were deduced from the top 10 primary metabolites which accounted for more than 80% of the total metabolites. The PLS-DA model of intestinal metabolome analysis was performed on TC pigs and BT pigs at **(B)** the pre-weaning stage, **(C)** the weaned stage, and **(D)** the growth stage.

**Figure 10:**
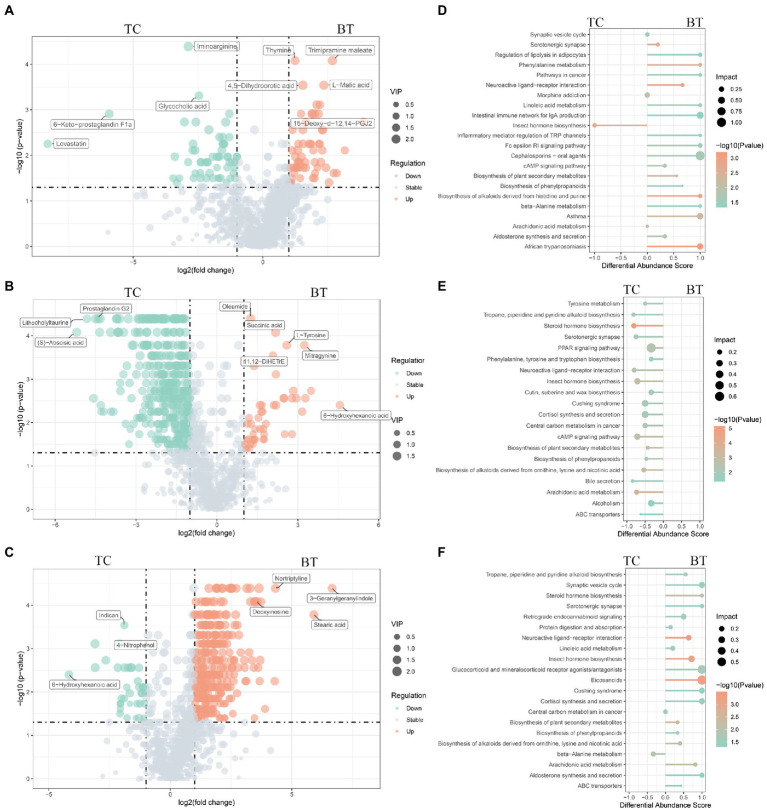
Different metabolites and metabolic pathways between TC pigs and BT pigs. Volcano plot of the differential metabolites at **(A)** the pre-weaning stage, **(B)** the weaned stage, and **(C)** the growth stage. Each point represents a metabolite, the points highlighted in green are enriched metabolites of TC pigs while those in red are enriched metabolites of BT pigs, and the size of the point represents the variable importance in projection. Differential metabolic pathways are grouped by **(D)** pre-weaning stage, **(E)** weaned stage, and **(F)** growth stage, the color represents the *p,* and the size of the point represents the impact value.

Metabolite composition, through untargeted metabolomics analysis (Supplementary Table S4), revealed significant pattern differences between TC pigs and BT pigs at various stages (Figures 9B–D), suggesting that the hybridization altered the metabolic processes of TC pigs. In addition, the class of carboxylic acids and derivatives presented the largest proportion of these primary metabolites (TCPW: 52.2%, BTPW: 51.4%, TCWD: 48.5%, BTWD: 40.2%, TCGT: 54.7%, and BTGT: 54.5%, Figure 9A). Moreover, the class of hydroxy acid and derivatives showed significant differences at the weaned stage and growth stage. However, the significantly different metabolites were fatty acids and steroids, including prostaglandins and short-chain fatty acid-related metabolites (Figures 10A–C). These data reflected that hybridization was associated with intestinal inflammation and development.

**Figure 11:**
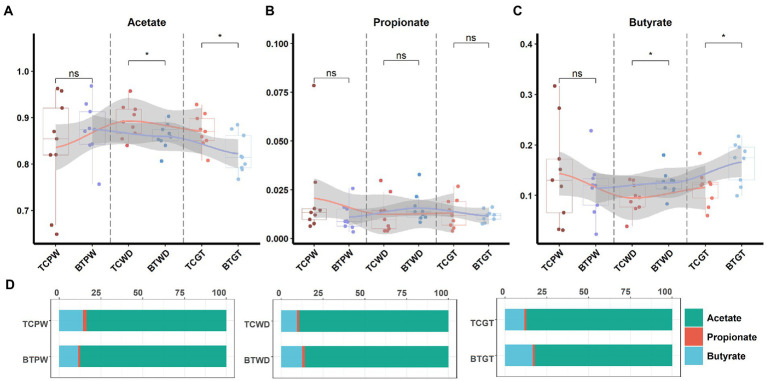
The tendency of acetate, propionate, and butyrate by stages: **(A)** the pre-weaning stage, **(B)** the weaned stage, and **(C)** the growth stage. Each point represents a sample, and differences in the figures were marked by “ns,” “*,” and “**” while no significance, *p* < 0.05 and *p* < 0.01, respectively. **(D)** Ratios of acetate, propionate, and butyrate by stage.

An important indication of microbial fermentation activity is the relative amount of acetate, propionate, and butyrate. They play an important role in maintaining intestinal function and integrity as well. Here, the proportions of acetate to propionate to butyrate in all the samples were in general close, and acetate had taken the most (Figure 11D). However, hybridization decreased the proportion of acetate (Figure 11A) and increased the proportion of butyrate (Figure 11C). Despite their similar intestinal histology, this indicates that the elevated acetate levels are more likely a function of the composition of the microbial community than that of intestinal histology.

### 3.7. Differences in the metabolic pathway between TC pigs and BT pigs over time

The functions of these changed metabolites were determined by the KEGG pathway analysis (Supplementary Table S5). The “arachidonic acid metabolism”, “intestinal immune network for IgA production”, “linoleic acid metabolism”, and “phenylalanine metabolism” were enriched after hybridization at the pre-weaning stage (Figure 10D). Similarly, the “PPAR signaling pathway” was also not enriched after hybridization at the weaned stage (Figure 10E), indicating that lipid metabolism and adipocyte differentiation may be downregulated by the hybridization. In addition, the pathway of “bile secretion” was impaired after hybridization at the weaned stage, which increased the risk of intestinal inflammation because bile acid was required for efficient lipid absorption and possessed powerful direct and indirect antimicrobial functions in the small intestine. Notably, many inflammation-related pathways were enriched after hybridization at the growth stage, including “eicosanoids”, “linoleic acid metabolism”, “arachidonic acid metabolism”, “cortisol biosynthesis and secretion”, and “steroid hormone biosynthesis” (Figure 10F). Nevertheless, “protein digestion and absorption” was still enriched in BT pigs of hybrid, reflecting its advantage on nutrient metabolism but potential inflammation.

**Table 1:** Andonis results of the microbiome.

	Pre-weaning		Weaned		Growth stage	
Factor	*R* ^2^	*p*	*R* ^2^	*p*	*R* ^2^	*p*
Species	0.12949	0.0119 *	0.2137	0.0015 **	0.15189	0.035 *
Gender	0.07567	0.1729	0.03536	0.6829	0.02192	0.8529
Maternal effect	0.07114	0.1884	0.02275	0.8879	0.06399	0.3063

*p < 0.05; **p < 0.01.

The influences of maternal effect and gender on the microbiome and metabolome were tested using the permutational analysis of variance (PERMANOVA) implemented using the Adonis function in the R “vegan” package with the Bray–Curtis method to calculate pairwise distances and 9,999 permutations. As a result, microbiome was little influenced by gender and maternal effect in TC pigs and BT pigs (Table 1). But the maternal effect had influenced the metabolome at pre-weaning stage (Table 2). Overall, differences in microbiome and metabolome was mainly caused by the different species.

**Table 2:** Andonis results of the metabolome.

	Pre-weaning		Weaned		Growth stage	
Factor	*R* ^2^	*p*	*R* ^2^	*p*	*R* ^2^	*p*
Species	0.33864	0.0001**	0.39841	0.0006**	0.41957	0.0005**
Gender	0.03111	0.3794	0.01146	0.08790	0.02761	0.5096
Maternal effect	0.38491	0.0225*	0.25339	0.6373	0.2561	0.4993

*p < 0.05; **p < 0.01.

### 3.8. Correlation analysis between microbiome and metabolism

**Figure 12:**
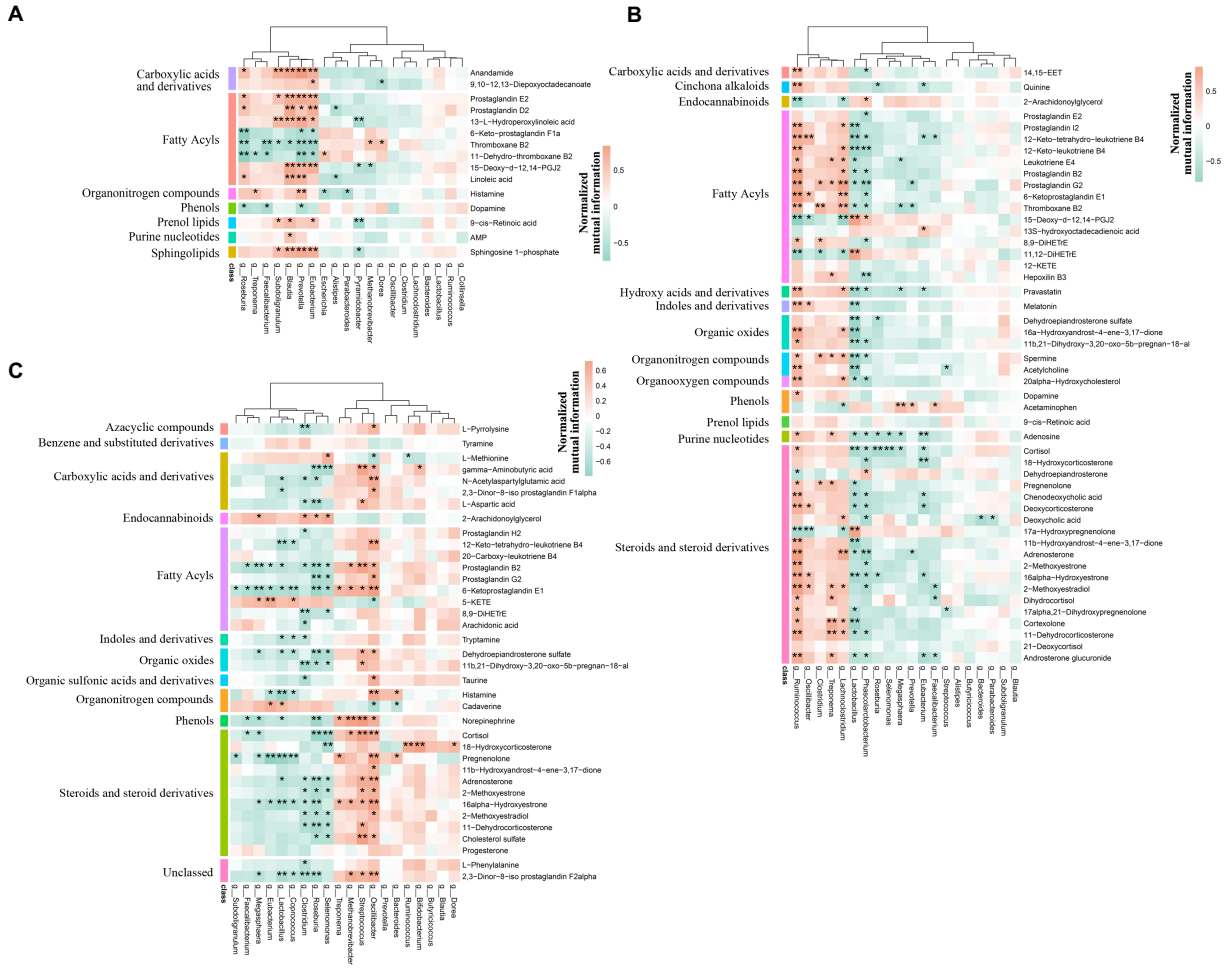
Spearman’s correlation analysis of microbiome and metabolism. The correlation analysis was performed by stages, including **(A)** the pre-weaning stage, **(B)** the weaned stage, and **(C)** the growth stage. Bacteria were deduced from the top 20 genera which accounted for more than 80% of the microbial sequence reads, and metabolites were deduced from the different metabolic pathways by stages. The color represents the correlation coefficient. Only correlation coefficient beyond 0.6 and *p-*value below 0.05 were considered significant and displayed in bold in Supplementary Tables; in figures, ** and * denote, respectively, *p-*values below 0.01 and 0.05.

We first verified that maternal effect and gender did not impact the selected metabolites through PERMANOVA (Table 3). The correlation analysis was performed by calculating Spearman’s correlation coefficient and *p*-value, reflecting the correlations between intestinal microbiota and metabolites. Furthermore, the metabolites were chosen from the different metabolic pathways. Here, the data suggested that *Prevotella*, the dominant bacteria at the pre-weaning stage, was positively correlated with the prostaglandin metabolites, including Prostaglandin E2, Prostaglandin D2, and 15-Deoxy-d-12,14-PGJ2 (Figure 12A). These metabolites were linked with chronic inflammation. We, then, found out that the majority of the differential metabolites were classified as fatty acyls and steroids and steroid derivatives at the weaned stage. Most of these metabolites were correlated with *Ruminococcus* and *Lactobacillus* (Figure 12B). Specifically, *Lactobacillus* was negatively correlated with the inflammation-related and steroid hormone biosynthesis-related metabolites, while *Ruminococcus* played the opposite role. Meanwhile, both *Lactobacillus* and *Ruminococcus* showed statistical differences in TC pigs and BT pigs at the weaned stage, indicating that they may regulate the differential metabolic pathways. Finally, the classification showed that most differential metabolites were involved in fatty acyls and steroids and steroid derivatives at the growth stage. These metabolites were also related to the inflammatory pathways, including “arachidonic acid metabolism”, “linoleic acid metabolism”, and “steroid hormone biosynthesis”. Similarly, *Lactobacillus* showed a negative correlation with the inflammation-related metabolites, but *Oscillibacter* played the opposite role this time (Figure 12C). These results confirmed our suspicion that the intestinal environment between TC pigs and BT pigs was altered by microbial communities and revealed the anti-inflammatory effect of *Lactobacillus*.

**Table 3:** Andonis results of the differential metabolites.

	Pre-weaning		Weaned		Growth Stage	
Factor	*R* ^2^	*p*	*R* ^2^	*p*	*R* ^2^	*p*
Species	0.17555	0.0543	0.42717	0.0009**	0.44146	0.0002**
Gender	0.02382	0.6404	0.02463	0.5897	0.05959	0.1447
Maternal effect	0.41096	0.3216	0.21844	0.7439	0.23925	0.4338

*p < 0.05; **p < 0.01.

## 4. Discussion

In the current study, we revealed several significant differences in intestinal functions and intestinal microbiome and metabolome between TC pigs and BT pigs. Furthermore, we verified that microbiota was significantly associated with many metabolites through correlation analysis. In conclusion, we identified that the hybridization increased the growth performance and the capability of nutrient absorption and metabolism of TC pigs but weakened the intestinal barrier function and development, especially in the colon.

Notably, significant differences were found in the intestinal microbiome and metabolome over time. We detected that the abundance of *Prevotella* was increased after hybridization, which may benefit nutrient metabolism (Chen et al., 2021; Ren et al., 2021). On the other hand, *Lactobacillus*, as the most dominant genus in TC pigs at the growth stage, contributed to intestinal health (Moeller and Sanders, 2020), but its abundance was decreased by hybridization. Meanwhile, the variation tendency of *Lactobacillus* was changed as well. As for the short-chain fatty acid, the proportion of acetate to propionate to butyrate in all the samples was in general close, but hybridization increased the average molar of butyrate and decreased the average molar of acetate. We also found that, through correlation analysis, *Lactobacillus* was negatively correlated with several steroid hormone-related and inflammation-related metabolites. These metabolites included cortisol and leukotriene and prostaglandin, which participated in the differential KEGG pathways, such as “steroid hormone biosynthesis” and “arachidonic acid metabolism” (Samuelsson, 1991; Shin et al., 2019a; Wang et al., 2021; Del Castillo-Izquierdo et al., 2022). These observations support the potential regulation by the hybridization and the anti-inflammatory effect of *Lactobacillus*. Histologically, it was similar before and after hybridization. In addition, the expression of several nutrient transporters, such as GLUT2 and ZNT1, was increased after hybridization, and the growth performance also confirmed that the capability of nutrient and absorption was better in BT pigs (Pluske et al., 2018; Quan et al., 2019; Yaqoob et al., 2021). As for the intestinal barrier, we detected the mRNA expression of occludin, REG3G, and IL10 was decreased after hybridization, while the LPS concentration of serum was increased. This evidence reflected that the hybridization impaired the intestinal barrier function of TC pigs (Tabung et al., 2017; Rohr et al., 2020; Engevik and Engevik, 2021). Furthermore, several intestinal development and proliferation-related mRNAs, such as CDX2 and IGF1 (Bonhomme et al., 2003; Van Landeghem et al., 2015; Sun et al., 2017), were decreased in colon tissues, which was unfavorable for bacterial colonization. In summary, our results reflected that hybridization reshaped the intestinal microbiome, metabolome, and functions.

Remarkably, *Prevotella* and *Lactobacillus* were the dominant genera during our studies. It was interesting that *Lactobacillus* was abundant in both pigs, although hybridization decreased its relative abundance. Moreover, the role of *Lactobacillus* has been reported as gut protection (Reid, 1999; Wang et al., 2018; Zhang et al., 2018; Dawood et al., 2019; Mao et al., 2019), since its production of SCFAs contributes to intestinal permeability (Chen et al., 2018; Mao et al., 2019) and regulation of the microbial composition (Xia et al., 2020). On the other hand, multiple phenotypes of TC pigs were altered by the hybridization. Most studies used to explain such changes through genome (Fu et al., 2021; Wu et al., 2022). However, the intestinal microbiome and metabolome participated in a variety of pathophysiological processes as well (Pei et al., 2021). Herein, we detected *Prevotella*, which played a role in the metabolism of proteins, peptides, and amino acids (Chen et al., 2021; Ren et al., 2021), was abundant after hybridization and may explain its better growth performance. According to the correlation analysis, inflammation-related metabolites were negatively correlated with *Lactobacillus*. Therefore, we predicted that *Lactobacillus* decreased intestinal inflammation. This agrees with the fact that *Lactobacillus* regulated intestinal homeostasis and immunity (Foysal et al., 2020; Xin et al., 2020). In addition, the relative amount of acetate was decreased by hybridization, which was negative for intestinal health. However, the intestinal function analysis also demonstrated a similar result that the intestinal barrier was impaired. This evidence displayed the advantages and disadvantages of hybridization in TC pigs. In addition, *Lactobacillus* still occupied a large proportion (TC pigs, 28.8%; BT pigs, 11.0%) in the total intestinal microbiota at the growth stage, compared to the other commercial pigs. These phenomena enticed us to investigate the possible regulations of *Lactobacillus*. Here, we gave several conjectures to learn more about the alteration of the colonization of *Lactobacillus*. KEGG pathway of the metagenome showed that ‘tetracycline biosynthesis’ was enriched in TC pigs, such antibiotics affected microbial colonization, including *Prevotella* and *Lactobacillus* (Takahashi et al., 2006; Campedelli et al., 2019; Li et al., 2019; Greppi et al., 2020). On the other hand, several steroid-related metabolites were negatively correlated with *Lactobacillus*. Some studies confirmed that serum steroid hormone could alter the diversity of the intestinal microbiome as well (Tetel et al., 2018; Shin et al., 2019b). This evidence indicated that *Lactobacillus* may be medicated by steroid hormones. These conjectures reflected the potential mechanism of host–microbiome interactions which may be changed by hybridization.

Several reports have shown that hybridization improved the growth performance and lean percentage in Chinese native pigs (Yen et al., 1991; Hyun et al., 2001; Luo et al., 2018; Chen et al., 2021). Meanwhile, intestinal microbiota could spread stably through mother–infant interactions (Desselberger, 2018; Ferretti et al., 2018), which made the microbial composition special among species. However, seldom studies associated the intestinal microbiota with the hybridization effect, while microbiota plays a vital role in the host physiological process. This prompted us to explore the differences in intestinal environment between crossbred pigs and purebred pigs. The noteworthy observation was the abundance of *Lactobacillus* that occupied a very high proportion in TC pigs (28.8%) and BT pigs (11.0%) at the growth stage, which was significantly different from the western commercial pigs (Han et al., 2018; Lim et al., 2019; Luo et al., 2022). In general, *Lactobacillus* was the dominant intestinal microbiota before weaning, and its abundance would decrease with age. We speculated that such a trait in TC pigs was caused by the coevolution between host and intestinal microbiota because evidence showed that *Lactobacillus* was positively correlated with the environmental temperature, but further evidence is still needed to confirm such host–microbiome interactions. In conclusion, our results shed light on the effect of hybridization on the intestinal environment in TC pigs and provide a novel perspective to the study of host–microbiome interaction between purebred pigs and crossbred pigs.

## 5. Conclusion

Given the findings that hybridization altered the gut microbiome, metabolome and intestinal functions, it is possible to explain how the intestinal microbiota may affect the traits of TC and BT pigs, including growth performance, nutrient digestion and absorption, and immune response. Furthermore, we explored the potential mechanisms of host–microbiome interactions. Taken together, these findings presented new insights into the role of hybridization in the intestinal microbiome–metabolome correlation, providing a theoretical basis for future microbiota transplantation and pig breeding programs.

## Data availability statement

The data presented in the study are deposited in the Sequence Read Archive (SRA) database, accession number PRJNA951204.

## Ethics statement

The animal study was reviewed and approved by Institutional Animal Care and Use Committee of Zhejiang University. Written informed consent was obtained from the owners for the participation of their animals in this study.

## Author contributions

JH, YZ, and ZW contributed to conception and design of the study. HL contributed to the acquisition of the samples. JH organized the database and wrote the first draft of the manuscript. YX performed the statistical analysis. GH, CP, and PZ wrote sections of the manuscript. All authors contributed to manuscript revision, read, and approved the submitted version.

## Funding

The research was supported by the Hainan Province Science and Technology Special Fund (ZDYF2022XDNY238) and the Project of Sanya Yazhou Bay Science and Technology City (SCKJ-JYRC-2022-07 and SKJC-2020-02-007).

## Conflict of interest

HL was employed by Long Jian Animal Husbandry Company.

The remaining authors declare that the research was conducted in the absence of any commercial or financial relationships that could be construed as a potential conflict of interest.

## Publisher’s note

All claims expressed in this article are solely those of the authors and do not necessarily represent those of their affiliated organizations, or those of the publisher, the editors and the reviewers. Any product that may be evaluated in this article, or claim that may be made by its manufacturer, is not guaranteed or endorsed by the publisher.

## Footnotes

^1^ http://www.hmdb.ca

^2^ http://www.massbank.jp/

^3^ http://www.lipidmaps.org

^4^ https://www.mzcloud.org

^5^ http://www.genome.jp/kegg/
